# An Account of the Efficacy of a Salivation, in Curing Pulmonary Consumption, in a Letter from Dr. M'Dowell, of York Town, Pennsylvania, to Dr. B. Rush

**Published:** 1808-01

**Authors:** Maxwell M'Dowell

**Affiliations:** York


### *An Account of the Efficacy of a Salivation, in curing Pulmonary Consumption, in a Letter from* Dr. M'DOWELL, *of York Town, Pennsylvania, to* Dr. B. RUSH.

Dear Sir,

IF you should be of opinion that the following case of phthisis, which yielded to the powers of mercury, will contribute its mite towards establishing a confidence in a remedy, at present unpopular, you are at liberty to make what use of it you may think proper.

Mr. D. aged about thirty years, whose thorax is of a structure favourable for the production of pulmonic complaints, observed his health gradually declining for the last two years; he at length became so much alarmed as to send for me on the 10th of September last. I found him very much emaciated, and labouring under a distressing cough, with a moderate purulent expectoration. He had profuse night sweats; his appetite entirely gone; his pulse was small, frequent, and feeble. I left six of your "antimonial powders" with him. On my next visit to him he told me he could not take the powders, as they were so very unpleasant to his taste. I then furnished him with a few papers, each containing three grains of calomel, directing him to take the contents of a paper three times a day. I requested him to drink cold water, or water with a toast a toast of bread in it. On the 17th, he complained of soreness in his mouth; 19th, a copious ptyalism, and his cough removed; 25th, a concentration of disease in the glands of his mouth. By means of the officious interference of some *benevolent* old ladies, who lay claim to a large portion of *experience,* my patient now became dissatisfied with his sore mouth, and I believe was upon the point of dismissing me. I entreated him to consider that his cough had left him; that he had no night sweats, and that his appetite was considerably restored. I endeavoured to make him acquainted with the manner in which the sore mouth would remove his original disease. My arguments, however, were not sufficient to remove the fears which had been raised in his mind, and he insisted upon having something to cure his mouth. I gave him some flor. sulphur; but prescribed it in such small quantities as would not be likely to lessen the mercurial disease in a short time;—29th, he informed me that through the importunities of his female visitors, he applied to a physician in town, who gave him a wash to cure his mouth. He had applied the wash only once, till he began to reflect upon what I told him respecting the operation of the mercury. He now expressed a willingness to submit entirely to my directions, and let me determine when his mouth was to be healed. His mouth was something better, and I was apprehensive that the quantity of sulphur which he had taken would remove the mercurial disease before his lungs could be restored to a healthy state;—though he was willing to let his mouth remain in its present situation as long as I thought necessary, yet he would not permit me to increase its soreness.

October 7th. His mouth was well; he felt a slight pain in his breast for two or three days which then disappeared, and he has, ever since, been perfectly free from all pulmonic complaints. I requested him to procure a flannel shirt, and wear it the ensuing winter.

November 6th. He says he is at this time in better health, and more fleshy than he has been for seven years.

I am, &c.

*York, Nov. 6, 1805.* MAXWELL M'DOWELL.

